# Factors Associated with Condom Use among Male College Students in Wuhan, China

**DOI:** 10.1371/journal.pone.0051782

**Published:** 2012-12-18

**Authors:** Lu Long, Ting Yuan, Min Wang, Chuan Xu, Jieyun Yin, Chengliang Xiong, Sheng Wei, Shaofa Nie

**Affiliations:** 1 Department of Epidemiology and Biostatistics, School of Public Health, Tongji Medical College, Huazhong University of Science and Technology, Wuhan, China; 2 Department of Pathology, School of Basic Medical, Hubei University of Chinese Medicine, Wuhan, China; 3 Department of Nosocomial Infection Management, Tongji Hospital, Huazhong University of Science and Technology, Wuhan, China; 4 Institute of Family Planning, Tongji Medical College, Huazhong University of Science and Technology, Wuhan, China; Tehran University of Medical Sciences, Iran (Republic of Islamic)

## Abstract

**Background:**

Using condoms consistently could prevent unintended pregnancy among young people. This study highlights multiple domains of inﬂuence on condom use among male college students in China, including knowledge, attitudes, health services utility on condom use and reproductive health information sources.

**Methodology/Principal Findings:**

To identify factors associated with condom use in Chinese male college students, we examined a sample of 870 sexually experienced male students in seven colleges in Wuhan, China, 2009. 535 (61.5%) of 870 male students reported condom use during their most recent sexual encounter. Male students with steady partners were more likely to use condoms than students with casual partners (adjusted OR = 3.11, 95%CI 2.30–4.20). And positive attitudes toward contraceptive responsibility were associated with greater odds of condom use (adjusted OR = 1.40, 95%CI 1.02–1.92). Only 54(6.2%) and 83(9.5%) of respondents reported that free condoms and reproductive health counseling were available at the student health center. Providing free condoms and reproductive health counseling at the student health central were associated with increased condom use among college students (both *P*<0.05). In addition, students who gained reproductive health information mainly through websites, television and radio programs were more likely to use condoms than through school education (all *P*<0.05).

**Conclusions:**

Improving attitudes of male students toward contraceptive responsibility, providing proper reproductive health information through mass media and making free condoms and reproductive health counseling available in school may help increase condom use among college students in China.

## Introduction

Dramatic social changes were seen in China over the past few decades. Social and economic development has opened up the attitudes of young Chinese toward sexuality, particularly among college students [1]. Research on sexuality and sexual behavior of college students in China has shown that an increasing number of young people have premarital sex [1], [2]. Two studies in Beijing and Ningbo indicated that over 10% of students or their partners have history of unintended pregnant [2], [3]. Many studies have shown the males’ important role in reproductive health decision-making, especially among teen populations [4]–[8]. Although couples are considered to have equal say in contraceptive decision making, most decisions might still be made by males, in many parts of the world [9]. A better understanding about multi-dimensional inﬂuence on condom use among male students will help inform unintended pregnancy and sexually transmitted diseases (STDs) prevention efforts.

Previous studies on condom use investigated a variety of factors, including demographic and socioeconomic characteristics and factors related to context of care such as condom providing and contraceptive counseling [9]–[17]. These studies showed that predisposing characteristics, including age, higher socioeconomic status, steady partnerships, adequate reproductive health knowledge and positive attitudes toward contraceptives are associated with increased odds of condom use [10], [12]. Providing free condoms at the student health center has also been considered to be associated with increased odds of condom use among college students since the cost and purchasing restrictions are barriers to consistent condom use [13], [14]. Furthermore, substantial unintended pregnancies among undergraduates require convenient and targeted contraceptive education and counseling [2], [15]. The lack of both counseling and privacy with respect to counseling prevents many young people from seeking contraceptives [16], [17].

Media which replaced the school education has been recognized as the most important source of reproduction health knowledge in youth in China and it is also a practical and accessible way of delivering sexual health education to young people [18]–[20]. Some well-designed websites, books, TV shows for sex education in local language especially for youth has been designed and implemented in recent years in China. However, little is known about the effects of diverse information sources on the condom use in male college students with sex experience.

The purpose of this study was to identify potential factors associated with condom use among male college students. These factors may be useful to make policy for pregnancy prevention programs.

## Materials and Methods

### Ethics Statement

The researchers obtained consent from all participants involved in the study. The study was specifically approved by the Research Ethics Committee in Tongji Medical College, Huazhong University of Science and Technology, Wuhan, China. We provided detailed information on the study to eligible college students and included only those who consented to participate. We obtained written informed consent from all subjects.

### Data Source and Study Population

This study was conducted in Wuhan city, Hubei Province. The data in our study were obtained through multistage stratified cluster sampling. Primary sampling units (PSU) were schools in comprehensive university, medical colleges and art colleges which were defined by the college entrance examination brochures. The clusters are approximately of equal size in terms of area and population. The ratio of these three types of schools is 4∶2:1. We selected 4 schools in comprehensive universities, 2schools in medical colleges and 1 school in art colleges by using simple random sampling. For the second stage, we selected 4 to 5 classes in Grades 1 to 4 in every selected school by using simple random sampling (approximately 30 students in each class).All male students in these classes were recruited. Of the 6,687 respondents who were approached to complete the survey, 6,381 responded validly, giving a final valid response rate of 95.4%. Only male students who had been sexually active in a heterosexual relationship in the past six months and who responded to the question about contraceptive use were included in the analysis. Of the 6,381 respondents, 13.6% (870/6,381) met the requirements. Our analytic sample included 870 male students.

For the survey, students were asked to complete the questionnaire by themselves in classroom after a trained staff addressed a brief introduction and instruction. The questionnaire was completely anonymous and should take approximately 10 minutes to complete. Data were collected from November to December 2009.

The questionnaire comprised 45 questions covering demographics, sexual history and behaviors, attitudes and knowledge relating to reproductive health, the availability of health services and main information sources. The questions were developed through reviewing literature and revised by qualitative methods including in-depth interview with 28 students (four males from each school) and seven focus group meetings (five at each school). Then the questionnaire was pilot-tested in a group of 50 randomly selected male students in a school.

The reliability was evaluated by comparing results from two surveys with a 2-week interval in 110 male college students. More than two thirds of questions had Kappa statistics over 0.4 (all p<0.05). Cronbach’s alpha coefficient which was calculated to determine internal consistency of the scales in the questionnaire ranged from 0.73–0.86. Principal components analysis suggested a good fit and the internal design of the questionnaire. In the knowledge scale, two common factors explained 44.8% and 50.6% of the total variance; in the attitude scale, two common factors explained 50.1% and 46.5% of the total variance. One common factors in the availability of health service scale contributed 51.3% of the total variance.

### Measurements

#### Dependent variables

Our dependent variables were derived from question regarding male students’ condom use over the past 6 months: “During the past 6 months, have you used condoms to prevent pregnancy or sexually transmitted diseases at the most sexual event?”.

#### Individual variables, health services and main information sources

We measured age, type of most recent sexual relationship (steady and casual) and money spent per month in our study. A casual partner was self-identified as a single encounter person. A steady partner was defined by study participants as a sexual partner who he met on a regular basis.

For access to health services, we determined whether the schools provided condoms and reproductive health counseling for free to the male students.

We measured the sources through which respondents had access to reproductive health information using one multiple choice question. The answer choices were: (1) books (2) websites (3) TV and radio programs (4) friends and classmates (5) school education (6) family members.

Reproductive health knowledge:

The final questionnaire consisted of 8 questions about reproductive health knowledge. We divided these questions into reproductive physiology-related knowledge questions and contraceptive method-related knowledge questions (questions were showed in Table 1). Four answer choices were provided for each item. Answer choices of these questions were listed below. For question 1, they are “bladder”, “ovarian”, “breast” and “don’t know”. For question 2, they are “Through her arteries”, “through her small intestine”, “through her fallopian tubes” and “don’t know”. For question 3, they are “adrenal”, “testis”, “bladder”, “don’t know”. For question 4. they are “saliva”, “pre-cum”, “blood” and “don’t know”. For question 5, they are “100 percent”, “nearly 50 percent”, “nearly 100 percent” and “don’t know”. For question 6, they are “one”, “two”, “as many as is comfortable” and “don’t know”. For question 7, they are “soon after ejaculation before the penis goes too soft”, “as soon as the penis is no longer erect”, “before the penis is erect” and “don’t know”. For question 8, they are “more than once if they're washed”, “only once”, “more than once if it's with the same person” and “don’t know”. To compare our results to previous studies, we suggest dividing the data into those who scored above the mean and those below (adequate and inadequate group) [10].

**Table 1:** Descriptions for reproductive health knowledge among male college students (individual items and percent correct).

Question	Total	Nonuse	Use	*p*
**Reproductive physiology-related knowledge**				
1. The female internal reproductive organ	81.5%	80.9%	81.9%	0.719
2. How do women’s eggs travel from the ovaries to the womb	40.6%	42.1%	39.6%	0.471
3. The male internal reproductive organ	69.0%	67.8%	69.7%	0.543
4. Which of these contain sperm	39.5%	42.1%	37.9%	0.224
**Condom use-related knowledge**				
5. How effective are condoms at preventing pregnancy	61.7%	53.5%	66.8%	<0.001
6. How many condoms should be worn to best protect against pregnancy and transmission of STDs?	29.7%	30.7%	29.0%	0.703
7. After sex, when should condoms be taken off the penis?	50.5%	34.1%	60.7%	<0.001
8. How often should male condoms be used?	80.4%	77.6%	82.1%	0.108

#### Attitudes toward reproductive health

There are 8 questions about attitudes in the final questionnaire. We divided these questions into two categories, attitudes toward contraceptive responsibility and condom use (questions were showed in Table 2). Two answer choices were provided for each item, Answer choices of these questions were listed below. For question 1–4, they are “yes” and “no”. For question 5, they are “beneficial” and “harmful”. For question 6, they are “easy to use” and “difficult to use”. For question 7, they are “a safe method of birth control” and “a dangerous method of birth control”. For question 8, they are “moral” and “immoral”. To compare our results to previous studies, we suggest dividing the data into those who scored above the mean and those below (positive and negative group) [21].

**Table 2:** Descriptions for attitudes toward reproductive health (individual items and percent correct).

Question	Total	Nonuse	use	*p*
**Attitudes toward contraceptive responsibility**				
1. Should take responsibility to “take care” of preventing sexually transmitted diseases	73.6%	71.3%	75.0%	0.240
2. Should take responsibility to “take care” of fertility regulation	84.4%	79.1%	87.7%	0.001
3. Should take responsibility to “take care” of unexpected pregnancy	52.8%	56.1%	62.8%	0.050
4. Should take responsibility to “take care” of abortion	60.2%	49.0%	55.1%	0.075
**Attitudes toward condom use**				
5. Beneficial	94.8%	94.3%	95.1%	0.599
6. Easy to use	17.6%	20.0%	16.1%	0.139
7. A safe method of birth control	93.3%	94.0%	92.9%	0.515
8. Moral	88.7%	86.0%	90.5%	0.041

### Statistical Analysis

Data were processed by using EpiData 3.1 and analyzed using STATA 10.0 software (StataCorp LP, College station, TX: USA). Frequencies and means were calculated for sample demographics. We proposed a multilevel logistic regression (MLR) model for the binary response (equal to 1 for condom use, 0 for nonuse) in order to considerate that the individual probability of an outcome is dependent on both individual level variables as well as group variables of subjects [22]–[24]. Thus, the first-level variables included: age, relationship status, spending per month, knowledge scores, attitudes scores, availability of hearth services, main information sources, whereas school was considered as second-level variable.

To examine the fixed effects of individual level covariates, and the extent to which these variables explained the random effects, we ran three model specifications. We started with empty model including only random intercept in order to explore aggregation without any effect of covariates; then we gradually added the fixed variables to the model: model 1 included a wide-ranging set of individual characteristics(age, relationship status, spending per month, reproductive physiology-related knowledge, condom use-related knowledge, attitudes toward contraceptive responsibility, attitudes toward condom use) [25]. Model 2 was a full model which added the availability of health services and main information sources in the model. The fixed effects were presented as CORs (95%CI), and the random effects as school variance with standard error (SE). The median odds ratio (MOR) quantifies the variation between the schools by comparing two persons from two randomly chosen schools. Values of MOR are always ≥1, if it is 1 there is no school level variation. The proportional change in variance (PCV) was calculated to express the change in the cluster variance between models.

## Results

### Sample Demographics

As shown in Table 3, 535(61.5%) of 870 male students reported condom use at the most recent sexual event in the past 6 months. 289 (33.2%) of sexually active male students accessed to reproductive health information mainly through school education. Only 35(4.0%) of the participants received sex education mainly from classmates and friends. Approximately 54 (6.2%) and 83 (9.5%) of the male students reported that their school health centers provided condoms and reproductive health counseling for free.

**Table 3:** Distribution of dependent variables and individual reproductive health knowledge, attitudes, information sources, and health services among sexually active male students.

	Estimate*
**Condom use**	
Yes	535(61.5)
No^a^	335(38.5)
**Age**	20.7±2.99
**Relationship status**	
Steady	517(59.4)
Casual	353(40.6)
**Spending per month**	600(500–900)
**Knowledge score**	
Reproductive Physiology	2.31±1.10
Contraceptive method	1.96±0.58
**Attitude score**	
Contraceptive responsibility	2.71±1.12
Condom use	2.93±0.91
**health services**	
Provision of condoms	54(6.2)
Provision of reproductive health counseling	83(9.5)
**Main information sources**	
School education	289(33.2)
Books	186(21.4)
Web site	179(20.6)
TV and radio programs	128(14.7)
Friends and classmates	35(4.0)
Family members	53(6.1)

* Data are number (%), mean±SD or median (IQR).

a including contraceptive nonuse and 5 types of contraception with >1% usage which were oral female contraceptives, withdrawal, vaginal hormonal ring, rhythm method and emergency contraceptives.

### Knowledge and Attitudes

Table 1 describes the percentages of correct response to each question on the reproductive health knowledge scale. More male students using condoms knew the effectiveness rate of condom and knew “when should condoms be taken off the penis?” than students reported no condom use (both *P*<0.001). Table 2 describes the percentages of positive responses to each item on the attitude scale. More male students using condoms reported that men should take responsibility to take care of fertility regulation and unexpected pregnancy than students reported no condom use (*P = *0.001 and *P* = 0.050, respectively). More male students using condoms reported using a condom for fertility regulation is a moral method than students reported no condom use (*P = *0.041).

### The Multilevel Model

Table 4 shows results from multilevel logistic regression analyses. Variance of the random effect of school clustering variable was highly significant in all models. Model 1 showed significant promoting effects of the wide-ranging set of individual characteristics on condom use among college students. As shown in model 1, factors associated with condom use included older age, steady sexual relationships, and positive attitudes toward contraceptive responsibility.

**Table 4:** Associations between individual reproductive health knowledge, attitude factors, health services, main information sources and condom use among sexually active male students.

	Nonuse	Use	Empty model	Model 1^a^	Model 2^b^
Variable	Freq (%)	Freq (%)		OR (95%CI)	OR (95%CI)
**Age**					
<20	147(43.9)	182(34.0)		Reference	Reference
≥20	188(56.1)	353(66.0)		1.51(1.09–2.10)*	1.41(1.00–1.99)
**Relationship status**					
Steady	145(43.3)	372(69.5)		2.91(2.17–3.90)***	3.11(2.30–4.20)***
Casual	190(56.7)	163(30.5)		Reference	Reference
**Spending per month**					
<600	172(51.3)	277(51.8)		1.02(0.76–1.37)	1.05(0.77–1.43)
≥600	163(48.7)	258(48.2)		Reference	Reference
**Knowledge score**					
**Reproductive physiology**					
0–2	174(51.9)	307(57.4)		Reference	Reference
3–4	161(48.1)	228(42.6)		1.01(0.69–1.48)	0.95(0.64–1.41)
**Condom use**					
0–1	72(21.5)	86(16.1)		Reference	Reference
2–4	263(78.5)	449(83.9)		1.35(0.92–1.97)	1.26(0.85–1.87)
**Attitude score**					
**Contraceptive responsibility**					
0–2	141(42.1)	175(32.7)		Reference	Reference
3–4	194(57.9)	360(67.3)		1.53(1.13–2.08)**	1.40(1.02–1.92)*
**Condom use**					
0–2	104(31.0)	129(24.1)		Reference	Reference
3–4	231(69.0)	406(75.9)		1.26(0.91–1.74)	1.20(0.86–1.68)
**Health service**					
**Provision of condoms**					
Yes	15(4.5)	49(9.1)			1.98(1.08–3.61)*
No/I don’t know	320(95.5)	486(90.9)			Reference
**Provision of counseling**					
Yes	26(7.7)	67(12.5)			1.54(1.08–2.74)*
No/I don’t know	309(92.3)	468(87.5)			Reference
**Main information sources**					
School education	114(34.1)	175(32.6)			Reference
Books	65(19.3)	121(22.7)			1.53(0.97–2.43)
Web site	67(19.9)	112(21.0)			1.64(1.02–2.70)*
TV and radio programs	62(18.4)	66(12.4)			2.31(1.10–4.84)*
Friends and classmates	12(3.6)	23(4.2)			1.61(0.98–2.65)
Family members	15(4.5)	38(7.1)			1.91(0.84–4.35)
**Random effects**					
School variance(SE)			2.156(0.031)***	1.311(0.042)***	1.156(0.056)***
MOR^c^			4.30	3.50	2.06
PCV^d^			Reference	39.19%	11.82%

Note. OR odds ratio, SE standard error, MOR median odds ratio, PCV proportional change in variance.

a Empty model adjusted for age, relationship status, spending per month, knowledge score and attitudes score.

b Model 1 adjusted for provision of condoms, provision of reproductive health counseling, main information sources,

c MOR quantifies cluster heterogeneity in terms of odds ratios.

d PCV expresses the change in the cluster variance between the empty model and model.1, model 1 and model 2, respectively,

* *P*<0.05,

** *P*<0.01,

*** *P*<0.001.

Model 2 (full model) evidenced the same exposure effects and significant effects of availability of health services and main information sources. We found that male students whose most recent sex partners were steady partners had three times the odds of condom use as male students whose most recent sex partners were casual partners (OR = 3.11, 95%CI 2.30–4.20). And male students who had positive attitudes toward contraceptive responsibility had 40% higher odds of condom use than those who had negative attitudes (OR = 1.40, 95%CI 1.02–1.92). Male students who reported that free condoms were provided in school had 98% higher odds of condom use than others (OR = 1.98, 95%CI 1.08–3.61), and free reproductive health counseling was provided by student health centers had 54% higher odds of condom use than others (OR = 1.54, 95%CI 1.08–2.74). In addition, compared to male students who accessed to reproductive health information mainly through school education, male students who accessed to reproductive health information mainly through websites had 64% higher odds of condom use (OR = 1.64, 95%CI 1.02–2.70), and male students who accessed to reproductive health information mainly through TV and radio programs had two times the odds of condom use (OR = 2.31, 95%CI 1.10–4.84).

MORs decreased to 3.500 and 2.062, whereas PVCs did to 39.19% and 11.82% for model 1 and 2, respectively (Table 4). The wide-ranging set of individual characteristics explained 39.19% of the variance. Controlling for the individual characteristics, availability of health services and main information sources explained another 11.82% of the between school variance in the co-occurrence of risks. Median odds ratio of 2.06 showed that some variation remained unexplained.

## Discussion

Male partner can improve reproductive health outcome by using condoms consistently with sexual partner to prevent HIV/STD and unintended pregnancy. A better understanding about factors associated with condom use among male students will not only protect students from unintended pregnancy but also help to reduce the high prevalence of HIV/STD among college students [26]. This study expanded upon previous studies to identify a wide-range set of individual characteristics associated with condom use among male college students. All of these factors may potentially benefit to improve male college students’ role in increasing condom use in sexual relationships. We also calculated that availability of health services and main productive health information sources explained over one-tenth of the condom use differences in the clustering of these factors after controlling for individual factors. These findings suggested that these factors should also be considered in the future policy making.

In our study, male college students whose most recent sex partner was steady partner were more likely to use condoms than individuals whose most recent sex partner was casual partner. This supported previous studies finding that students in a relationship were more likely to use contraceptives than those are single, possibly because of the association between reduced communication in early casual relationships and risky sexual behaviors [27]–[29]. In contrast, a study by Manlove et al. found that shorter sexual relationships were associated with increased condom use and consistency among male adolescents, because many males and females in this relationship do not know their partners’ sexual history [10]. Future longitudinal research is needed to determine associations between sexual relationship and subsequent condom use.

In our study, only 17.6% of students reported that condom is easy to use. Similar to our study, previous studies also found that most of male students thought condoms difficult to use although they were aware of reproductive health knowledge [30], [31]. Some studies suggested that condom-use knowledge and skills were needed to make healthy and informed decisions with greater condom use [11], [32]. However, we did not find any significant association between adequate contraceptive method-related knowledge and condom use among male college students in the full model.

As hypothesized, positive attitudes toward contraceptive responsibility were associated with greater odds of condom use, supporting previous research [33]. It is possibly because students who shared the contraceptive responsibility are more likely to attend medical appointments with partners and remind each other to use condoms on a daily basis [33]. In addition, shared responsibility is also helpful to share the financial cost of contraception, while cost-sharing for contraceptives would more equitably save costs and improve the consistency of contraceptive use [33], [34].

As hypothesized, we found that providing free condom at student health centers was associated with increased odds of condom use among male college students. It indicated that services for contraceptive provision could be improved and policies modified in order to facilitate access to contraceptive care [35], [36]. Incorporating these services into health protection programs could benefit students as college-aged students have high rates of unintended pregnancies and high risk of STDs [37]. We also found that provision of reproductive health counseling among students at risk for unintended pregnancy or HIV/STD was associated with greater condom use among them. This finding supported previous research which stated that contraceptive counseling may increase contraceptive use and reduce the number of unintended pregnancies, particularly if the counseling is personalized to the individual’s contraceptive needs [38], [39].

In our study, Medias like Internet, TV and radio programs were the main sources of contraceptive knowledge for young men except the school education. And we found that the students who gained information mainly through mass media like Internet, TV and radio programs were more likely to use condoms than through school education, whereas reproductive health education from school is recognized as the most basic tool to increase a young person’s knowledge and capacity for self-protection [18]. This finding supported the previous research which stated that Internet is a practical and accessible way to deliver sexual health education to youths [12], [40], [41]. An Internet-based intervention study conducted among college students in Shanghai, China, reported that providing sex education to students through the Internet was feasible and effective [18]. This is possibly because using social media tools such as internet can facilitate immediate access to reproductive health information, and reinforce the importance of communication between partners about sexual risks and condom use [42]. We also found that the students who gained information mainly through TV and radio programs were more likely to use condoms than through school education. Previous research in Bangladesh even suggested that TV program was an important determinant factor of condom use and preferred method [42]. These findings highlight the demand for rich media services focusing on the young or covering important reproductive health information young people need.

The findings of our study were limited by several factors. First, this study represented a small proportion of male college students in Wuhan. One previous study showed that students who did not take part in our project are in fact more affected than those who participate [43]. Students having more open views on sexuality may be more likely to complete a survey. Therefore, our findings may not be representative of the general student population. Second, condom use information was reported by students retrospectively, whereas condom use would ideally be measured by using daily calendars. Finally, no cause-effect relationship was observed in our study for the limitation of the cross-sectional design. Although these limitations may exist, our findings extended previous research by identifying many domains of inﬂuence on condom use among male college students.

In conclusion, we found that positive attitudes toward contraceptive responsibility were associated with condom use. To prevent unintended pregnancy and HIV/STD, reproductive health education should focus on not only increasing the knowledge about contraception but also improving attitudes toward contraceptive responsibility. And male college students should be encouraged to use well-designed, local-language websites and TV programs. We also proposed that better availability of condoms and contraceptive counseling played an important role in increasing condom use by directly addressing the needs of male students. We believe that our study will contribute to a better understanding of what kind of sex education and reproductive health education tool should be provided, and will guide interventions design and public policies making to improve health services. Further research is needed to evaluate condom use and determine how the factors mentioned in this study affect condom use.
